# Ergovaline stability in tall fescue based on sample handling and storage methods

**DOI:** 10.3389/fchem.2014.00076

**Published:** 2014-09-08

**Authors:** Krista Lea, Lori Smith, Cynthia Gaskill, Robert Coleman, S. Ray Smith

**Affiliations:** ^1^Department of Plant and Soil Sciences, University of KentuckyLexington, KY, USA; ^2^Veterinary Diagnostic Laboratory, Department of Veterinary Science, University of KentuckyLexington, KY, USA; ^3^Department of Animal and Food Science, University of KentuckyLexington, KY, USA

**Keywords:** tall fescue, *Neotyphodium coenophialum*, ergot alkaloids and ergovaline, transportation and storage, HPLC with fluorescence detection

## Abstract

Ergovaline is an ergot alkaloid produced by the endophyte *Neotyphodium coenophialum* (Morgan-Jones and Gams) found in tall fescue [*Schedonorus arundinacea* (Schreb.) Dumort.] and blamed for a multitude of livestock disorders. Ergovaline is known to be unstable and affected by many variables. The objective of this study was to determine the effect of sample handling and storage on the stability of ergovaline in tall fescue samples. Fresh tall fescue was collected from a horse farm in central Kentucky at three harvest dates and transported on ice to the University of Kentucky Veterinary Diagnostic Laboratory. Plant material was frozen in liquid nitrogen, milled and mixed before being allocated into different sub-samples. Three sub-samples were assigned to each of 14 sample handling or storage treatments. Sample handling included increased heat and UV light to simulate transportation in a vehicle and on ice in a cooler per standard transportation recommendations. Storage conditions included storage at 22°C, 5°C, and −20°C for up to 28 days. Each sub-sample was then analyzed for ergovaline concentration using HPLC with fluorescence detection and this experiment was repeated for each harvest date. Sub-samples exposed to UV light and heat lost a significant fraction of ergovaline in 2 h, while sub-samples stored on ice in a cooler showed no change in ergovaline in 2 h. All sub-samples stored at 22°C, 5°C, and −20°C lost a significant fraction of ergovaline in the first 24 h of storage. There was little change in ergovaline in the freezer (−20°C) after the first 24 h up to 28 days of storage but intermittent losses were observed at 22°C and 5°C. To obtain results that most closely represent levels in the field, all samples should be transported on ice to the laboratory immediately after harvest for same day analysis. If immediate testing is not possible, samples should be stored at −20°C until analysis.

## Introduction

Tall fescue is a cool season perennial grass native to Europe and well adapted to much of the United States, particularly in the southeast where it covers an estimated 35 million acres of livestock pasture, hay fields and roadways (Ball et al., 2007). Most tall fescue plants in the southeastern US are known to be infected with an endophyte, *Neotyphodium coenophialum* (Morgan-Jones and Gams) that has formed a mutualistic relationship with the plant. The endophyte lives in the intercellular spaces of the plant (Bacon and Siegel, 1988) and produces many compounds. Some of these compounds benefit the plant and result in increased drought and disease tolerance (Arachevaleta et al., 1989; Gwinn and Gavin, 1992). However, the endophyte also produces a range of alkaloids toxic to foraging animals.

Ergovaline, a strong vasoconstrictor, is the major ergopeptine alkaloid produced by the endophyte and is believed to be the primary cause of fescue toxicosis in livestock (Lyons et al., 1986; Belesky et al., 1988; Klotz et al., 2007). Extensive research has suggested that ingestion of endophyte infected tall fescue plants by cattle results in increased core body temperature (Aldrich et al., 1993), decreased average daily gain (Hopkins and Alison, 2006), rough hair coats (McClanahan et al., 2008), and decreased weaning weights (Peters et al., 1992) that result in an annual loss of $600 million in the US beef industry (Hoveland, 1993). Pregnant horses often experience prolonged gestation (Monroe et al., 1988), difficulty foaling (Putnam et al., 1991), low milk production (Kosanke et al., 1987), and decreased breeding efficiency (Brendemuehl et al., 1994). Due to the economic impacts of endophyte infected tall fescue on livestock, monitoring ergovaline in tall fescue from livestock pastures is becoming common for research and farm situations.

Ergovaline in solution is known to be unstable due to isomerization, hydrolysis and sensitivity to light, air, acids, and bases (Garner et al., 1993). However, very little information is known about the effects of sample handling. The objectives of this research are to evaluate the stability of ergovaline in tall fescue material stored over time under various conditions and to establish ideal sample handling and transportation recommendations.

## Materials and methods

All samples were gathered from a single pasture on a horse farm located near Lexington, KY on the following dates: 1 May 2012, 21 August 2012, and 11 June 2013. The pasture was ~0.4 ha in size and contained primarily cool season grasses including tall fescue, Kentucky bluegrass (*Poa pretensis* L.), and orchardgrass (*Dactylis glomerata* L.). The pasture was seeded with orchardgrass and Kentucky bluegrass in the fall of 2011 to increase vegetative cover. Due to the recent overseeding and an excessive distance from the barn, this paddock was ungrazed by horses throughout the entire sampling period, but was routinely mowed to ~12.5 cm.

The pasture had been tested several times for tall fescue endophyte infection from 2009 through 2013, and the infection rates ranged from 80 to 100%. Endophyte infection levels were established by collecting ~20 tillers from 10 locations throughout the field for endophyte testing at the University of Kentucky Regulatory Services Laboratory (immunoblot endophyte test kits, Agrinostics, Athens, GA).

Ergovaline quantitation had also been performed periodically on tall fescue found in this pasture and was observed to consistently contain more than 500 ppb ergovaline during the growing season from 2008 to 2012.

### Harvest and sampling for ergovaline analysis

On day 0 of each harvest period, ~2 kg of fresh tall fescue was harvested using a rice knife at 5–7.5 cm above the soil surface. Plant material was placed on ice and transported immediately to the laboratory (less than 1 h). Once at the laboratory, the sample was cut into 2.5–7.5 cm lengths, frozen using liquid nitrogen and milled while frozen. Milling was performed using a Stein Mill (Steinlite Corporation, Atchison, KS). Milled material was then mixed by hand and separated into 42 individual sub-samples, each ~5 g. Sub-samples were placed into 120 ml whirl-pak bags and immediately placed into their assigned treatments with three sub-samples assigned to each treatment. Average time from harvest to treatment application was 2.5–4 h and samples were kept chilled or frozen the entire time.

### Treatments

Treatments were designed to simulate real world situations (Table 1). The Control treatment was placed in the freezer (−20°C) for 2 h before sample preparation began. The Ice treatment was designed to simulate a sample that had been collected by a producer or county agent and transported to the laboratory on ice. This was accomplished by placing the appropriate sub-samples in a cooler on ice for 2 h. Similarly, the Light + Heat treatment was designed to simulate the sample transported inside a vehicle under hot and sunny weather conditions. These sub-samples were exposed to 38 ± 1.5°C and UV light by placing them under full spectrum bulbs (three GE Residential Ecolux 40W bulbs, three Hatch Lighting F40T12/D/DX 40W bulbs and one 60 watt incandescent bulb) for 2 h. The entire apparatus was covered with vinyl and aluminum foil to contain heat. Storage treatments included storage at ambient temperature (22°C), refrigerator (5°C), and freezer (−20°C) temperatures for varied amounts of time. These various storage locations and durations were meant to simulate storage at a testing laboratory. On the day of analysis, samples were removed from their treatment locations and set on the lab bench for less than 10 min before sample preparation began.

**Table 1:** **List of treatments for sample handling and storage**.

**Treatment abbreviation**	**Treatment**	**Time of treatment**	**Temperature (°C)**
C	Control	2 h	−20
I	Ice	2 h	1
LH	Light + Heat	2 h	38.5
A1	Ambient temp	1 Day	22
A2	Ambient temp	2 Days	22
A3	Ambient temp	3 Days	22
R1	Refrigerator	1 Day	5
R3	Refrigerator	3 Days	5
R6	Refrigerator	6 Days	5
F1	Freezer	1 Day	−20
F7	Freezer	7 Days	−20
F14	Freezer	14 Days	−20
F21	Freezer	21 Days	−20
F28	Freezer	28 Days	−20

### Quantitation by HPLC and fluorescence detection

The analytical method for this experiment was as adapted from Spiering et al. (2002) at the University of Kentucky Veterinary Diagnostic Laboratory for research and diagnostic purposes to assess total ergovaline (ergovaline + ergovalinine) content in tall fescue.

Ergovaline and ergovalinine are stereoisomers that are often present in solution. Degree of epimerization from ergovaline to ergovalinine depends on many variables including type of extraction solvent, amount of light and heat to which the solution is exposed and the amount of time the ergot alkaloid has been dissolved in solution (Smith and Shappell, 2002). Ergotamine and its epimer, ergotaminine, are susceptible to the same variables.

#### Chemicals and reagents

Ergotamine tartrate was purchased in solid, purified form (>97%) from Sigma-Aldrich (St. Louis, MO, USA). Ergovaline tartrate was obtained as a custom synthetic product (>93%) from F. T. Smith (Pharmaceutical Sciences, Auburn University, Auburn, AL, USA). Ammonium acetate, methanol, acetronitrile, and 2-propanol (HPLC-grade) were purchased from Fisher Scientific (Fair Lawn, NJ, USA). D,L—lactic acid (85%) was purchased from Acros Organics (New Jersey, USA). Purified water was prepared using a Mega-Pure MP-6A distillation system and an EASYpure II RF water conditioner (Barnstead, Dubuque, IA, USA).

For each sub-sample, 1.00 ± 0.25 g of plant material was placed in a 15 ml disposable conical centrifuge tube and the exact mass was recorded; 4950 μL of internal extraction solution [50% aqueous 2-propanol/1% (v/v) lactic acid] was added, as well as 50 μL 10 μM ergotamine. Ergotamine was used as an internal standard because it is structurally and chemically similar to ergovaline, therefore losses of ergotamine through the sample preparation process are assumed to be similar to ergovaline losses. The sub-sample was then vortexed for ~30 s to completely wet the plant material with extraction solvent. All samples analyzed concurrently were then placed on a mixer/rotator [Multi Mixer and Rotator (UNICO MTR22), United Products & Instruments, Inc.], covered with aluminum foil to minimize exposure to light and rotated for 1 h. Samples were centrifuged for 12 min at 3000 rpm. The supernatant was removed, passed through a PVDF syringe filter (0.45 μm, 25 mm diameter) to remove particulates and dispensed into silanized, amber 2-mL autosampler vials.

For each day of analysis, four standard solutions with increasing ergovaline concentrations ranging from 0 to 270 ppb and a reagent blank were prepared and analyzed. The standard solutions all contained 58.2 ppb ergotamine. The reagent blank was prepared using an empty 15 mL centrifuge tube, adding all solutions as if the tube contained a sample and treated it as a sample throughout the analysis.

#### Moisture content

A separate ~1 g portion of plant material from each sub-sample was weighed, dried for 24 h at 100°C in a forced-air drying oven (Isotemp Oven, Thermo Fisher Scientific, Waltham, MA, USA) and weighed again to determine moisture content of the original sample. Moisture content of each sub-sample was determined on the date of analysis to correct for the moisture contribution to the total subsample mass.

#### Chromatography and fluorescence analysis

Reverse-phase chromatographic separation of ergovaline, ergotamine and their respective epimers from fescue matrix components was achieved using a U-3000 dual pump high pressure liquid chromatography (HPLC) system equipped with a RF-2000 fluorimeter (Thermo Fisher Scientific, Waltham, MA, USA). The mobile phase was delivered at a constant flow rate of 1.0 mL/min. The instrument was equipped with an Acclaim 120 C18 HPLC Analytical Column (3 μm particles, 120 Å; 4.6 mm ID × 100 mm, Thermo Fisher Scientific, Waltham, MA, USA), preceded by a Security Guard Column Cartridge System (C18; 4 mm × 3.0 mm; Phenomenex, Torrance, CA, USA). The column compartment was set and maintained at 30°C throughout the analysis. Binary mobile phases consisted of 75% (v/v) 0.1 M ammonium acetate in acetonitrile (mobile phase A) and 25% (v/v) 0.1 M ammonium acetate in acetonitrile (mobile phase B), each of which was degassed by helium sparging for ~10 min prior to use. Sample extracts and standard solutions (20 μL) were injected into the initial gradient conditions of 95% mobile phase A/5% mobile phase B. Immediately following injection the mobile phase B was increased at a linear rate to 20% over the next 20 min, then further increased to 50% over the next 15 min and finally increased to 70% over the next 5 min. The gradient profile was then held at 30% mobile phase A/70% mobile phase B for 7 min to wash the analytical column of any residual nonpolar matrix components. At 47 min after injection, the initial gradient conditions were resumed and the column was re-equilibrated in preparation for the next injection. Fluorescence detection parameters included excitation and emission wavelengths set to 310 and 410 nm, respectively, and each sample required 52 min to run. The autosampler tray was covered throughout analysis to prevent light exposure.

Calibration curves were produced by plotting the instrument response against the ergovaline concentration of each standard solution. For this method, the instrument response was defined as the peak area ratio of the total ergovaline detected (e.g., the sum of the peak areas for ergovaline and ergovalinine) to the total ergotamine detected (e.g., the sum of ergotamine and its epimer). A linear regression was fit to the standard data and, using the resulting trendline equation, the total ergovaline concentration was calculated for each fescue sample. The final result was corrected for moisture content and the reported values are presented on a dry matter basis. The term “ergovaline” in all results will refer to ergovaline + ergovalinine.

### Statistics

All results were analyzed in SAS 9.3 using the general linear models and least-squares means procedures (PROC GLM and lsmeans). A significant difference in ergovaline concentrations was observed across harvests, therefore treatments were compared within the same harvest only and considered significant at *P* < 0.05.

## Results

**Figure 1:**
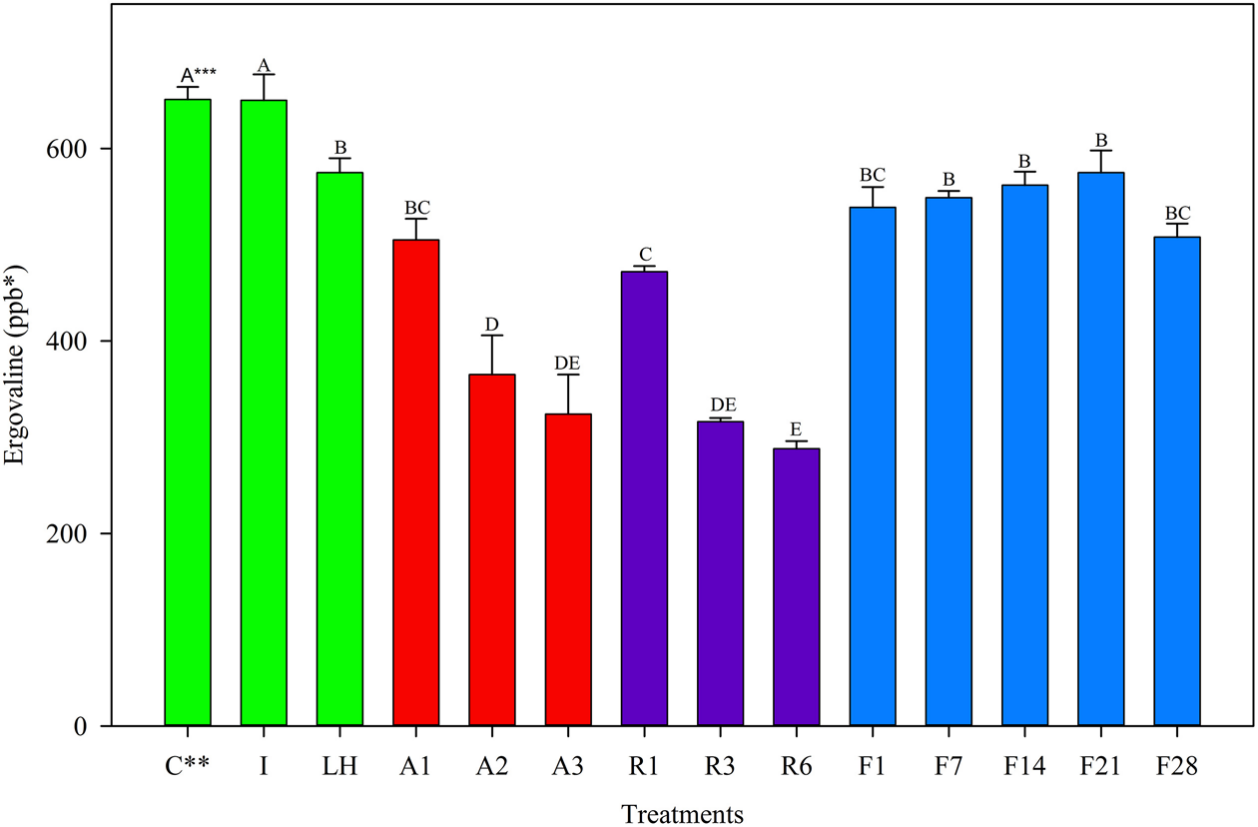
**Ergovaline concentration of all tall fescue sub-samples in various storage conditions from May 2012 harvest**. ^*^Parts per billion. ^**^Treatment abbreviations are as follows: C, Control; I, Ice; LH, Light + Heat; A1, Ambient Day 1; A2, Ambient Day 2; A3, Ambient Day 3; R1, Refrigerator Day 1; R3, Refrigerator Day 3; R6, Refrigerator Day 6; F1, Freezer Day 1; F7, Freezer Day 7; F14, Freezer Day 14; F21, Freezer Day 21; and F28, Freezer Day 28. ^***^Matching letters indicate no statistical difference at a significance level of *P* < 0.05.

For the May 2012 harvest, no difference in ergovaline concentration was observed between the Ice treatment and the Control (Figure 1); Samples exposed to light + heat for 2 h experienced significant losses in ergovaline. Samples stored at 22°C, 5°C, and −20°C for 24 h contained less ergovaline compared to the control (22, 27, and 17%, respectively), (Table 2). Losses experienced in the first 24 h were not different from one another, regardless of storage temperature. Ergovaline concentrations did not vary throughout 28 days of −20°C storage in May 2012.

**Table 2:** **Ergovaline concentrations after 24 h of storage at various temperatures for three harvests**.

	Ergovaline (ppb^*^)					
	**May 2012**		**August 2012**		**June 2013**	
Control	651	^A^†^^	1606	^A^	524	^A^
Ambient (22°C)	505	^B^	638	^B^	394	^B^
Refrigerator (5°C)	472	^B^	648	^B^	386	^B^
Freezer (−20°C)	539	^B^	693	^B^	423	^B^

* Parts per billion.

† Matching letters within a column indicate no statistical difference (p < 0.05) within a single harvest.

**Figure 2:**
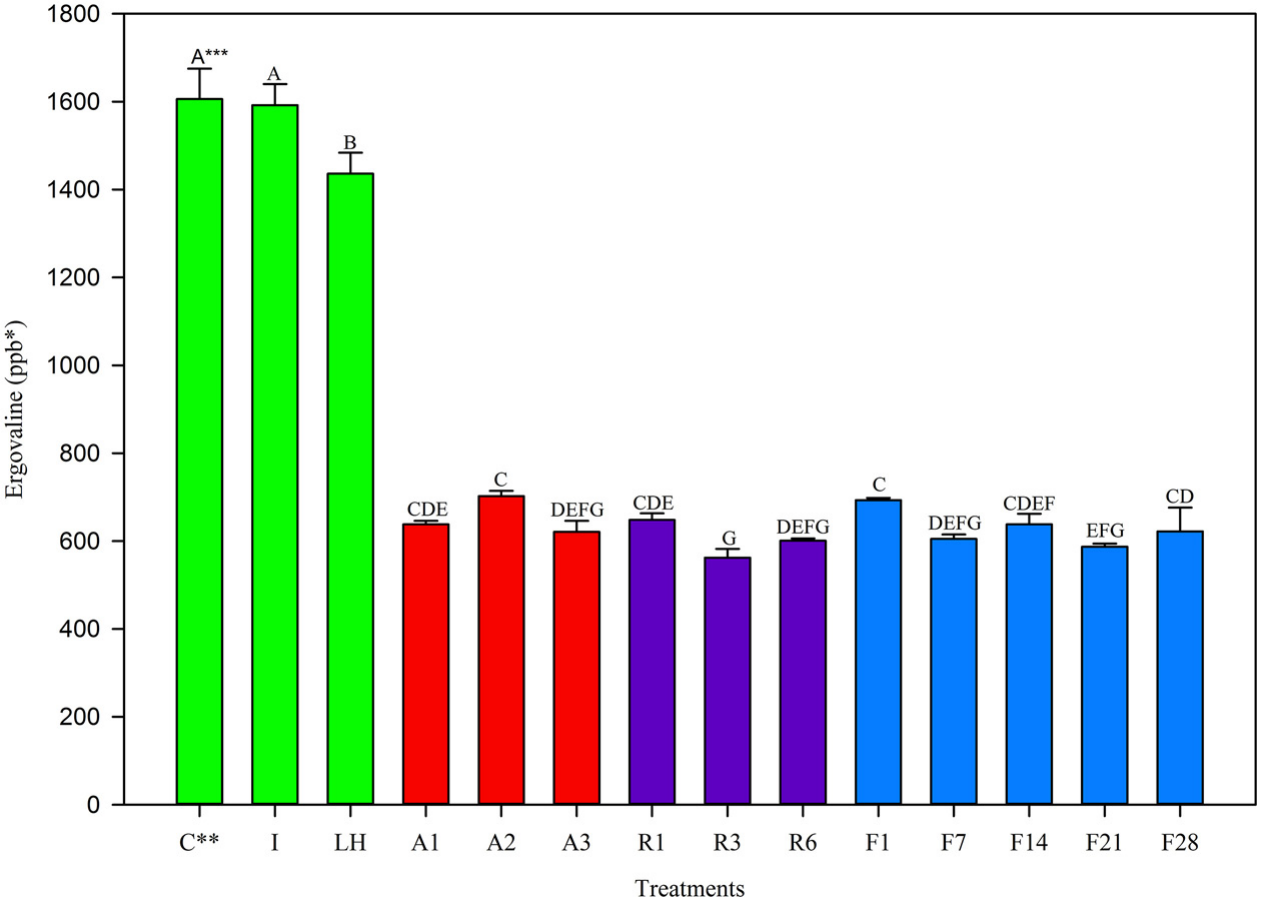
**Ergovaline concentration of all tall fescue sub-samples in various storage conditions from August 2012 harvest**. ^*^Parts per billion. ^**^Treatment abbreviations are as follows: C, Control; I, Ice; LH, Light + Heat; A1, Ambient Day 1; A2, Ambient Day 2; A3, Ambient Day 3; R1, Refrigerator Day 1; R3, Refrigerator Day 3; R6, Refrigerator Day 6; F1, Freezer Day 1; F7, Freezer Day 7; F14, Freezer Day 14; F21, Freezer Day 21; and F28, Freezer Day 28. ^***^Matching letters indicate no statistical difference at a significance level of *P* < 0.05.

In the August 2012 harvest, similar observations were made to May 2012. The Control and Ice treatment showed equivalent ergovaline concentration (Figure 2) while the light + heat lost significant ergovaline. Storage losses in the first 24 h (Table 2) were significant from the control, and not different from one another (60% at 22°C and 5°C, 57% at −20°C). Little variation in ergovaline concentration was observed over 28 days of −20°C storage.

**Figure 3:**
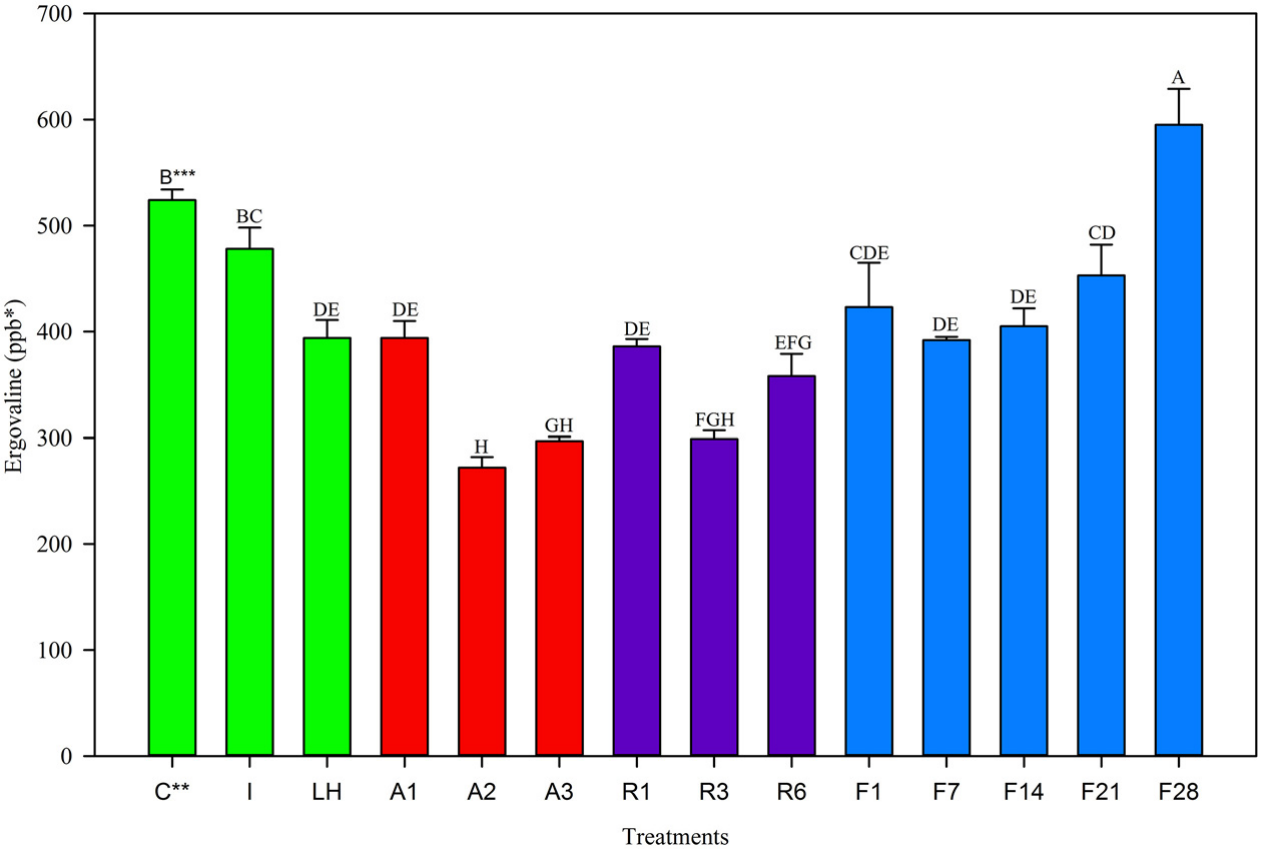
**Ergovaline concentration of all tall fescue sub-samples in various storage conditions from June 2013 harvest**. ^*^Parts per billion. ^**^Treatment abbreviations are as follows: C, Control; I, Ice; LH, Light + Heat; A1, Ambient Day 1; A2, Ambient Day 2; A3, Ambient Day 3; R1, Refrigerator Day 1; R3, Refrigerator Day 3; R6, Refrigerator Day 6; F1, Freezer Day 1; F7, Freezer Day 7; F14, Freezer Day 14; F21, Freezer Day 21; and F28, Freezer Day 28. ^***^Matching letters indicate no statistical difference at a significance level of *P* < 0.05.

Trends in the June 2013 harvest were similar to the two previous harvests. The Control and Ice treatment were not different from one another (Figure 3), however the light+heat was reduced. Twenty-four hour storage losses were significantly different than the Control but not different from one another and were 25% at 22°C, 26% at 5°C, and 19% at −20°C (Table 2). No change in ergovaline concentration was observed in samples stored at −20°C for 21 days. Interestingly, ergovaline concentration was higher at day 28; laboratory procedures were carefully reviewed to insure that this anomaly was not related to sample preparation or calculation errors.

## Discussion

These results indicated that ergovaline in fresh plant material that had been frozen and milled was not consistently stable. In all three harvests, there was no significant loss of ergovaline in the first 2 h when the sub-samples were kept on ice in a cooler, compared to the control. However exposure to heat and UV light resulted in a significant loss after 2 h for all three harvests. This suggested that during transportation, sample handling is an important factor. Samples for research and diagnostics should be transported on ice in a cooler to the testing laboratory as soon as possible.

Ergovaline concentrations were reduced in all material in the first 24 h of storage at all storage temperatures. For each harvest, losses observed at −20°C were not different from losses observed at 5°C or 22°C. This suggests that in the first 24 h, ergovaline is not stable in harvested material. Few laboratories have the capabilities to analyze tall fescue samples the same day as harvest, therefore it is likely that ergovaline levels in most research and diagnostic samples are reported lower than what is in the field at the time of harvest.

Twenty-four hour losses observed in May and June harvests when stored at −20°C were similar in magnitude (17 and 19%, respectively) compared to the August harvest (57%). It is possible that ergovaline stability in the plant is dependent on the time of year. However, the initial concentrations of the control treatments for the May and June harvests (651 ppb in May 2012, 524 ppb in June 2013) were also more similar to one another than the initial concentration for the control treatment in August 2012 (1606 ppb). This suggests that the instability of ergovaline may be related to the concentration of ergovaline in the plant material at harvest: material with higher concentrations may show a greater loss in the first 24 h than material with lower concentration. Norman et al. (2007) also observed higher percent loss of ergovaline in hay bales with higher initial concentrations than those with lower concentrations at harvest.

After a significant decrease in ergovaline concentration in the first 24 h of storage, ergovaline concentrations changed very little over 28 days of storage at −20°C in all three harvests. This suggests that samples can be stored for up to a month without further degradation. If steps are taken to reduce or account for losses in the first 24 h, then laboratories will be able to store samples for up to at least 1 month before analysis and achieve accurate test results.

It is unclear what effect milling may have on ergovaline before storage. Allocation of whole tillers to treatment groups was attempted, but large differences in ergovaline concentrations (up to 34%) were observed between groups before any storage treatment was applied. This is similar to what Mace and Baker (2012) reported, observing 68% variability in ergovaline concentration between plants and 32% variability between tillers within a plant. It is unclear if milling had any effect on the concentrations throughout this study, but milling was conducted before the application of the storage treatments to ensure homogenous mixing of the plant material. Controlled studies of the effect of milling would be valuable in the future.

## Conclusion

In conclusion, sample handling and storage conditions have a significant impact on the ergovaline concentrations within the tall fescue plant material. To obtain the most accurate analysis, all samples should be transported on ice to the laboratory immediately after harvest for same day analysis. If immediate testing is not possible, samples should be stored at −20°C until analysis.

Based on the results of this research, ergovaline measurements may be significantly lower after 24 h of storage, regardless of storage temperature. Most testing facilities do not have the capacity to guarantee testing the same day as harvest. Therefore researchers, extension personnel, veterinarians and producers should assume that testing results are significantly lower than actual levels in pasture.

### Conflict of interest statement

The authors declare that the research was conducted in the absence of any commercial or financial relationships that could be construed as a potential conflict of interest.
